# A new index of cortical plasticity induced by paired associative stimulation to describe cognitive status in aged healthy subjects

**DOI:** 10.3389/fphys.2025.1625137

**Published:** 2025-09-24

**Authors:** Nicola Loi, Francesca Ginatempo, Mohammed Zeroual, Lucia Ventura, Antonella Cano, Carmen Oneto, Paola Ortu, Maria Rita Piras, Franca Deriu

**Affiliations:** ^1^ Azienda Ospedaliero Universitaria di Sassari, Sassari, Italy; ^2^ Department of Biomedical Sciences, University of Sassari, Sassari, Italy; ^3^ National Research Council – Institute for Genetics and Biomedical Research, Sassari, Italy; ^4^ Unit of Endocrinology, Nutritional and Metabolic Disorders, Azienda Ospedaliero Universitaria di Sassari, Sassari, Italy

**Keywords:** transcranial magnetic stimulation, paired associative stimulation, cognitive skills, aged subjects, paired associative stimulation responsiveness

## Abstract

**Introduction:**

Cortical plasticity is a key factor for cognitive skills, and paired associative stimulation (PAS) is useful to study it in humans. Currently, due to the number of non-responders to PAS and discrepancies in the post-PAS time-points assessed, a plasticity index describing PAS effects and correlating it to cognitive status is lacking. Therefore, this study investigated which PAS index better discriminates between responders (RRs) and non-responders (NRs) and correlates with cognitive status.

**Methods:**

Seventy-six healthy aged subjects (67.0 ± 7.2 y.o., 35 males) were enrolled. The Montreal Cognitive Assessment (MoCA), the Mini-Mental State Examination (MMSE), and the Addenbrooke’s Cognitive Assessment (ACER) were used to assess cognitive status. Motor-evoked potentials (MEPs) were recorded from the first dorsal interosseous muscle at baseline and after 0, 10, 20, and 30 min from PAS, pairing peripheral median nerve stimulation with a transcranial magnetic stimulation stimulus over the left primary motor cortex. MEP amplitude was used to calculate the grand average (GrA), which is the mostused PAS plasticity index, along with two newly introduced indexes: the curve concavity (CC) and the pre- vs. post-PAS difference (PPPD). CC described the curve shape of the PAS effects, while PPPD calculated the significant differences between the baseline and post-PAS MEP amplitude.

**Results:**

CC demonstrated good consistency as PAS-plasticity index with high odds ratios and sensibility in the discrimination of responsiveness to PAS; PPPD had higher specificity in the identification of RRs. Only the MoCA score was significantly higher (p = 0.006) in RRs than in NRs when the two groups were discriminated according to CC, and it significantly correlated with CC (p = 0.013).

**Discussion:**

In conclusion, CC may represent a potential PAS-plasticity index to describe the cortical plasticity and cognitive status in humans, with a possible practical application in patients with cognitive impairment.

## 1 Introduction

Synaptic plasticity is a physiological mechanism in which activity-dependent modifications of the strength or efficacy of synaptic transmissions occur after exogenous or endogenous stimulation (Citri and Malenka, 2008). This mechanism is crucial to adapt to environmental changes, and it is the basis for learning phenomena (Kandel et al., 2014). Among several types of synaptic plastic changes, long-term potentiation (LTP) is a key mechanism that is able to strengthen specifically stimulated synaptic connections, requiring high-frequency stimulation and near-synchronous activation of both pre- and post-synaptic neurons (Malenka and Bear, 2004; Kandel et al., 2014).

In humans, LTP at the cortical level can be studied through a transcranial magnetic stimulation (TMS) protocol named paired associative stimulation (PAS) (Stefan et al., 2000; 2002; Carson and Kennedy, 2013). PAS is administered by pairing electrical stimulation (ES) of the peripheral nerve afference followed by a TMS stimulus over the primary motor cortex (M1), for 100–200 times, determining a rapidly evolving (<30 min) and long-lasting (>60 min) increase in cortical excitability assessed through motor-evoked potentials (MEPs) (Stefan et al., 2000; 2002). This increase in excitability is because of the LTP-like mechanism (Stefan et al., 2000; Wolters et al., 2003), which depends on the enhanced strength of the connections between the primary somatosensory cortex (S1) and the excitatory interneurons within M1 (Stefan et al., 2000; 2002; Ridding and Taylor, 2001). Several studies demonstrated that PAS improves motor learning in healthy subjects as well as in pathological conditions (Ziemann et al., 2004; Wessel et al., 2015). However, there is no evidence demonstrating a significant correlation between PAS-related gain and cognitive status in healthy subjects (Schättin et al., 2018; Minkova et al., 2019).

In the last 24 years, several studies have used PAS effects as a clinical parameter for studying cortical plasticity, with conflicting results obtained when dealing with patients with cognitive decline (Battaglia et al., 2007; Terranova et al., 2013; Lahr et al., 2016; Minkova et al., 2019; Di Lorenzo et al., 2020; Meder et al., 2021). Some studies found differences in PAS effects between cognitively impaired patients and healthy controls (Battaglia et al., 2007; Terranova et al., 2013; Di Lorenzo et al., 2020), while others failed to detect any significant effect (Lahr et al., 2016; Minkova et al., 2019; Meder et al., 2021). These conflicting results could be because of the different experimental procedures used. In fact, some studies observed PAS effects at only one time point after PAS administration (Tecchio et al., 2008; Fathi et al., 2010), while others observed the effects at two (Cirillo et al., 2009; Singh et al., 2014; Lahr et al., 2016; Schättin et al., 2018; Meder et al., 2021) or even more than two time points after PAS delivery (Müller-Dahlhaus et al., 2008; Terranova et al., 2013; Bhandari et al., 2018; Minkova et al., 2019).

Another factor that affects PAS results is the subjects’ responsiveness to the PAS protocol. It has been observed that PAS elicited the expected effects in 60% or less of the participants (Karabanov et al., 2016; Lahr et al., 2016; Minkova et al., 2019), and it depends on the subjects’ inter- and intra-variability due to circadian fluctuations, time of the day (Sale et al., 2007), alertness (Kamke et al., 2012), attentional state (Stefan et al., 2004), stimulation intensity (Müller-Dahlhaus et al., 2008), genetic traits (Cheeran et al., 2008), cortical thickness (List et al., 2013), and microstructural properties of the white matter (Klöppel et al., 2008). This variable response to the PAS protocol limits its use as a tool that can be correlated with the subject’s cognitive status, especially in aged people and patients with cognitive decline. For this reason, it is necessary to determine a plasticity index that is able to describe the whole PAS effects, including all the time points, and discriminate between responders (RRs) and non-responders (NRs) to PAS. In the literature, the most-used PAS-plasticity index is the grand average (GrA) (Wolters et al., 2005; Müller-Dahlhaus et al., 2008; Lahr et al., 2016; Minkova et al., 2019), which is the average of the post-PAS MEP amplitudes. More studies tried to relate GrA with the cognitive status in humans, but no significant correlation was found (Schättin et al., 2018; Minkova et al., 2019), raising the question of whether it is the most suitable PAS-plasticity index.

Therefore, we aimed to identify PAS-plasticity indexes that are able to describe the PAS effect across several time points and correlate it with the cognitive status. For this reason, two PAS-plasticity indexes were defined as the curve concavity (CC) and the pre- vs. post-PAS difference (PPPD), and these indexes were compared with the GrA.

Both CC and PPPD were established considering that the plasticity effect is a long-lasting phenomenon that is recordable at different time points after PAS administration. This consideration differs from several works that identified the responsivity to the PAS protocol based on the increase in the MEP amplitude at only one time point after PAS, which could be the result of changes in the cortical excitability rather than the synaptic plasticity phenomenon (Malenka and Bear, 2004; Kandel et al., 2014). In this light, the mean MEP amplitudes at each time point were used to determine the curve, and its concavity was used as a PAS-plasticity index. In addition, the PPPD, which was determined when at least two post-PAS time points showed a significant increase in MEP amplitude compared to that at baseline, was taken as another PAS-plasticity index. Both CC and PPPD were used to discriminate between RRs and NRs to the PAS protocol.

Finally, we verified whether these PAS-plasticity indexes correlated with cognitive status. In the perspective of clinical application of these indexes in patients with cognitive decline, the study involved a group of aged, healthy subjects since it has been demonstrated that cognitive decline is an age-related feature and can occur even before the older age threshold (Murman, 2015).

## 2 Methods

### 2.1 Participants

Seventy-six healthy, aged subjects (mean age 67.0 ± 7.2; range: 50 years–89 years old; 35 males), all right-handed according to the Oldfield inventory scale, participated in the study. According to the age range (10 years), the sample consisted of the following age groups: 50–59 = 13 subjects, 60–69 = 35 subjects, 70–79 = 24 subjects, and 80–89 = 4 subjects. Informed written consent was obtained from all the subjects. The experimental procedures were approved by the Local Ethics Committee (Sardinia Ethics Committee Prot. PG/2023/5172, 06/04/2023) and were conducted in accordance with the guidelines of the Declaration of Helsinki. None of the participants had a history and/or current signs/symptoms of neurological and/or psychiatric diseases, and none used psychotropic drugs (neuroleptics and anticonvulsive medications). The exclusion criteria followed the TMS safety guidelines (Rossi et al., 2021). Subjects were seated in a comfortable chair, and they were asked to stay relaxed but alert during the recordings, which were performed in a quiet room.

### 2.2 Cognitive evaluation

The cognitive status was evaluated using standardized neuropsychological tests including the Montreal Cognitive Assessment (MoCA) (Nasreddine et al., 2005), the Addenbrooke’s Cognitive Examination revised (ACE-R) (Mioshi et al., 2006), and the Mini-Mental State Examination (MMSE) (Folstein et al., 1975). Moreover, subcategory scores of MoCA and ACE-R were also used as variables. MoCA included the assessment of visuo-spatial abilities, executive functions, language, orientation, attention, and memory (raw scores). ACE-R included the assessment of orientation, memory, verbal fluency, language, and visuo-spatial abilities. Tests were administered and analyzed by expert neuropsychologists. Raw values were corrected for age and education level.

### 2.3 Electromyography (EMG)

Surface electromyography (EMG) was recorded from the first dorsal interosseous (FDI) muscle using 9-mm-diameter Ag–AgCl surface electrodes. The active electrode was placed over the muscle belly, the reference electrode was placed over the metacarpophalangeal joint of the second finger, and the ground electrode was placed over the forearm (Ginatempo et al., 2022). EMG signals were recorded (D360 amplifier, Digitimer Ltd., Welwyn Garden City, UK), amplified (1,000 times), filtered (bandpass 3 Hz–3,000 Hz), and sampled at 5 kHz using a 1,401 power analog-to-digital converter and Signal 6 software (Cambridge Electronic Design, Cambridge, UK).

### 2.4 Transcranial magnetic stimulation (TMS)

TMS was performed using a 70-mm figure-of-eight coil connected to a Magstim 200 stimulator through a BiStim module (Magstim Co., Whitland and Dyfed, UK). The optimal stimulation site for the right FDI muscle was carefully identified and then marked with a soft-tip pen on the scalp to maintain the same coil position throughout the experiments. The handle of the coil was pointed posteriorly and laterally, at approximately 45° to the interhemispheric line (Rossini et al., 2015). The resting motor threshold (RMT) was measured as the lowest TMS intensity that is able to elicit MEPs of 0.05 mV in the relaxed muscle in at least five out of 10 consecutive trials. RMT was expressed as a percentage of the maximum stimulator output (MSO). The test stimulus intensity was set at 120% of RMT.

### 2.5 Paired associative stimulation (PAS)

Experiments were performed in the afternoon (between 15:00 and 17:00). The PAS intervention was administered by pairing 200 electrical stimulation (ES) of the right median nerve with TMS over the left hand M1 using inter-stimulus intervals of 25 ms at 0.25 Hz (Stefan et al., 2000; 2002) lasting for approximately 13 min. ES was delivered to the right median nerve at the wrist through a pair of cup electrodes (cathode distal) connected to a current stimulator (model DS7; Digitimer Ltd). Single square-wave pulses (0.2-ms duration) were delivered, and the stimulus intensity was set at three times the perceptive threshold (PT). TMS was delivered over the left hand M1 using a stimulus intensity at 110% of RMT.

Fifteen MEPs were collected from the resting FDI before (baseline) and immediately (T0), 10 (T10), 20 (T20), and 30 (T30) minutes after PAS administration based on previous studies that showed the strongest PAS effect in this time-window (Wischnewski and Schutter, 2016). During PAS stimulation, subjects were asked to mentally count the stimuli to maintain attention. Effects of PAS were measured as MEPs’ peak-to-peak amplitude collected at each tested time interval. PAS-plasticity indexes were determined comparing the ratio of the post-PAS MEPs at each time-point with baseline MEPs.

### 2.6 Data analysis

Responsiveness to PAS was determined using GrA, CC, and PPPD. Since PPPD is not a continuous variable, only GrA and CC were used as PAS plasticity indexes for the correlation analysis with the scores in the cognitive batteries (MMSE, ACE-R, and MoCA) and their subcategories. Each subject was categorized as RR or NR according to the three PAS plasticity index criteria. They were divided into RR and NR based on the GrA. The CC criterion reclassified the same subjects differently, and the PPPD criterion applied its own way of grouping them, ensuring that the same 76 subjects were categorized as RR or NR on three separate occasions according to the three PAS plasticity index criteria.

**FIGURE 1:**
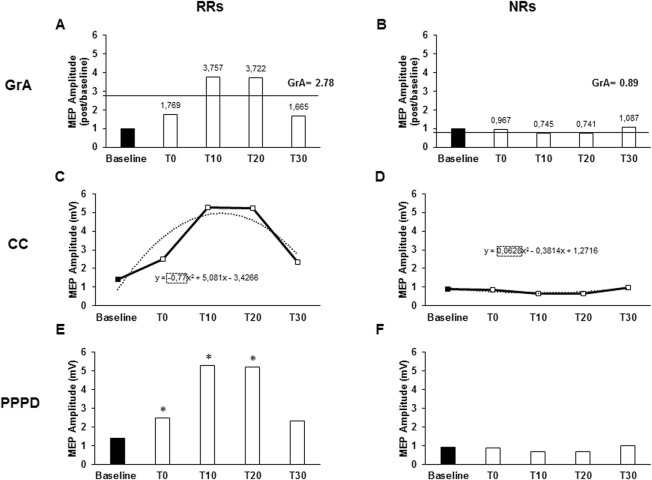
Responsiveness characteristics when using the grand average (GrA), curve concavity (CC), or pre- vs. post-PAS difference (PPPD) as PAS plasticity indexes. The charts describe the responsiveness of two representative subjects of a non-responder (NR) and a responder (RR) to the PAS protocol, assessed as the MEP amplitude at baseline and after 0 (T0), 10 (T10), 20 (T20), and 30 (T30) minutes from PAS delivery. Continued lines identify GrA of the post-PAS time point MEPs; GrA was calculated as the mean value of the MEP amplitude ratios calculated at each post-PAS time point (post-PAS time point/baseline). GrA >1 identified an RR **(A)**, while GrA <1 identified an NR **(B)**. Dashed lines identify the CC. CC was obtained using the polynomial function of the curve (y = ax2 + bx + c), where the “a” coefficient expresses the concavity of the curve. The curve function was built from the raw MEP amplitudes collected throughout the five time points. CC <0 identified a RR **(C)**, while CC >0 identified a NR **(D)**. In the PPPD, asterisks identify those time points where MEPs were significantly higher than baseline MEPs; PPPD identified RRs when post-PAS raw MEP amplitudes were significantly higher than baseline MEPs in at least two out of the four post-PAS MEPs **(E)**; if less than two time points were higher than the baseline, it determined NRs **(F)**. *p < 0.05.

GrA was calculated as the mean value of the MEP amplitude ratios calculated at each post-PAS time point (post-PAS time point/baseline) (Müller-Dahlhaus et al., 2008). Subjects were categorized as RRs when GrA >1 (Figure 1A) and as NRs when GrA <1 (Figure 1B).

The MEP amplitude curve was used to determine CC, which was obtained using the polynomial function of the curve *(y = ax* ^*2*^ *+ bx + c)*, where the “*a*” coefficient expresses the concavity of the curve. The curve function was built from the raw MEP amplitudes collected throughout the five time points (baseline, T0, T10, T20, and T30) using the Excel polynomial function (Office 365 pro plus, Microsoft Corporation, Redmond, USA). A negative *a* value (negative concavity) identified RRs (Figure 1C), while a positive *a* value (positive concavity) identified NRs (Figure 1D).

PPPD identified RRs when post-PAS raw MEP amplitudes were significantly higher than those of baseline MEP in at least two out of the four post-PAS MEPs (T0, T10, T20, and T30) (Figure 1E); otherwise, they were identified as NRs (Figure 1F). To describe responsiveness through PPPD, paired Student’s t-test was used to compare the means of baseline MEP amplitudes with the means of each post-PAS time point. Since PPPD is a categorical variable, it was not used in the correlation analysis.

### 2.7 Statistical analysis

Statistical analysis was performed with SPSS 26 software (SPSS Inc., Chicago, IL, USA). The analysis of variance (ANOVA) and planned *post hoc* t-test with Bonferroni correction for multiple comparisons were used. Compound symmetry was evaluated with Mauchly’s test, and Greenhouse–Geisser correction was performed when required. Results were considered statistically significant when the p-value was <0.05. A preliminary descriptive analysis was performed to evaluate the presence of outliers in all the variables (raw amplitude MEPs, MEP ratios, CC, GrA values, and MoCA, ACE-R and MMSE scores). Subjects were considered outliers when they exhibited extreme values for all TMS and/or all cognitive variables, which led to their exclusion from further analysis.

Moreover, to understand if cortical excitability could describe MoCA scores, we used a linear regression model with MoCA as the dependent variable and baseline MEP and RMT as the independent variables.

#### 2.7.1 Evaluation of PAS effects

Repeated-measure (RM) ANOVA was used to assess PAS effects using MEP amplitude as a variable with time (baseline, T0, T10, T20, and T30) as the within-subject factor. Moreover, to assess if the PAS effect was present within the groups of RRs and NRs, RM-ANOVA was separately performed for RRs and NRs, using MEP amplitude as a variable with time (baseline, T0, T10, T20, and T30) as the within-subject factor. Responsiveness was detected using GrA as the PAS plasticity index.

#### 2.7.2 Evaluation of the characteristics of the PAS plasticity indexes

To assess the goodness of fit between the modeled curve derived from the CC and the individual data points obtained at each time interval, a goodness of fit analysis using curve estimation regression on all raw MEP values (used to construct the CC curve) was performed for each subject. In this analysis, the dependent variable was MEP amplitude, and the independent variable was the MEP trial number at each time point (baseline, T0, T10, T20, and T30). Model fit was evaluated using the mean *R* ^2^ across the subjects and the root-mean-squared error (RMSE).

To understand if RRs and NRs were different regarding cortical excitability, once we determined the RR and NR groups based on each PAS-plasticity index (GrA, CC, and PPPD), one-way ANOVA with variables RMT and MEP at baseline was performed separately with the index (GrA, CC, and PPPD) as the between-subject factor. Chi-square analysis was carried out to assess whether a significant difference was present between the rate of RRs and NRs when discriminated with the three PAS-plasticity indexes (GrA, CC, and PPPD). The intra-class correlation coefficient (ICC) was calculated to assess the individual consistency of CC in describing the PAS plasticity effect in comparison to GrA. Inter-rater reliability analysis was conducted using a two-way mixed-effects model with a consistency type. In this context, the two indices—CC and GrA—were treated as “raters” for the ICC calculation. It is important to note that these indices do not represent repeated measurements of the same variable, but rather that of two distinct metrics.

A contingency model was used to calculate the likelihood of CC and PPPD in the identification of RRs. GrA, which represents the commonly used PAS plasticity index in the literature (Wolters et al., 2005; Müller-Dahlhaus et al., 2008; Lahr et al., 2016; Minkova et al., 2019), was compared to CC and PPPD regarding the responsiveness discrimination, calculating the odds ratios (ORs).

The contingency model of the receiver operating characteristic (ROC) (Zweig and Campbell, 1993) was used to calculate the sensibility and specificity in the PAS responsiveness characterization of CC and PPPD. GrA, which represents the commonly used PAS plasticity index in the literature (Wolters et al., 2005; Müller-Dahlhaus et al., 2008; Lahr et al., 2016; Minkova et al., 2019), was used as the reference value. A 2 x 2 contingency table was determined using GrA as the PAS plasticity index reference and comparing CC and PPPD in sensibility and specificity in the discrimination of responsiveness. True RRs were defined when both the GrA and CC/PPPD indexes described a plastic effect; on the contrary, true NRs were defined when both GrA and CC/PPPD indexes did not individuate a PAS effect. False RRs were defined as the subjects who were RRs for the CC/PPPD index but not for the reference index, i.e*.*, GrA; false NRs were defined as the subjects who were RRs for the reference index (GrA), but not for CC/PPPD. Sensibility [true RRs/(true RRs + false NRs)] and specificity [true NRs/(true NRs + false RRs)] were then calculated. In this light, sensibility describes the amount of wrongly considered NRs by the new indexes compared to the GrA; while, specificity describes the number of wrongly considered RRs by the new indexes compared to the GrA. False RRs and false NRs were not excluded by the following analyses.

Furthermore, in order to assess the robustness of sensibility and specificity of CC and PPPD as indexes discriminating between RRs and NRs, the same contingency table was developed in a reduced sample (70%) (Babyak, 2004).

#### 2.7.3 Assessment of the influence of gender and age on the neurophysiological tests and the PAS-plasticity indexes

To evaluate the effect of gender in cognitive tests (MMSE, ACE-R, and MoCA) and PAS plasticity indexes (GrA and CC), a one-way ANOVA was performed, while PPPD differences in gender frequency were assessed through chi-square analysis. To evaluate the influence of age on PAS effects and cognitive status, Spearman’s bivariate correlation analysis was performed to correlate age with cognitive scores (MMSE, ACE-R, and MoCA) and PAS plasticity indexes (GrA and CC), while for PPPD, a one-way ANOVA was used with index as a factor.

#### 2.7.4 Evaluation of the influence of responsiveness to the PAS protocol on the cognitive status

To assess if cognitive scores were different between RRs and NRs, a one-way ANOVA was separately performed for each cognitive test (MMSE, ACE-R, and MoCA) and their subcategories using the PAS plasticity indexes as a factor (GrA, CC, and PPPD).

Moreover, a Bayesian one-way ANOVA was performed to better understand differences between RRs and NRs in the cognitive scores based on the frequentist approach. The Bayesian factor (BF) was used, according to the Bayesian information criterion (BIC), to probabilistically test the null hypothesis (H0), i.e., no difference between RRs and NRs, while a significant difference between RRs and NRs identified the alternative hypothesis (H1). BF values were interpreted as follows: BF up to 0.33, evidence for H0; BF between 0.33 and 3.0, no evidence; and BF >3.0, evidence for H1. The strength of the evidence (anecdotal, moderate, strong, very strong, and extreme) was also appraised according to the same guidelines by Lee and Wagenmakers (2014)

### 2.8 Assessment of the correlation between PAS plasticity indexes and cognitive status

To select the appropriate test for the correlation analysis, the normality of the variable distributions was assessed using the Kolmogorov–Smirnov (K–S) and Shapiro–Wilk (S–W) tests. Variables were classified as non-normally distributed when both tests showed significant results (p < 0.05). Correlation analysis was conducted between cognitive scores (MoCA, ACE-R, and MMSE) and PAS plasticity indexes (GrA and CC).

## 3 Results

### 3.1 Evaluation of the PAS effects

**FIGURE 2:**
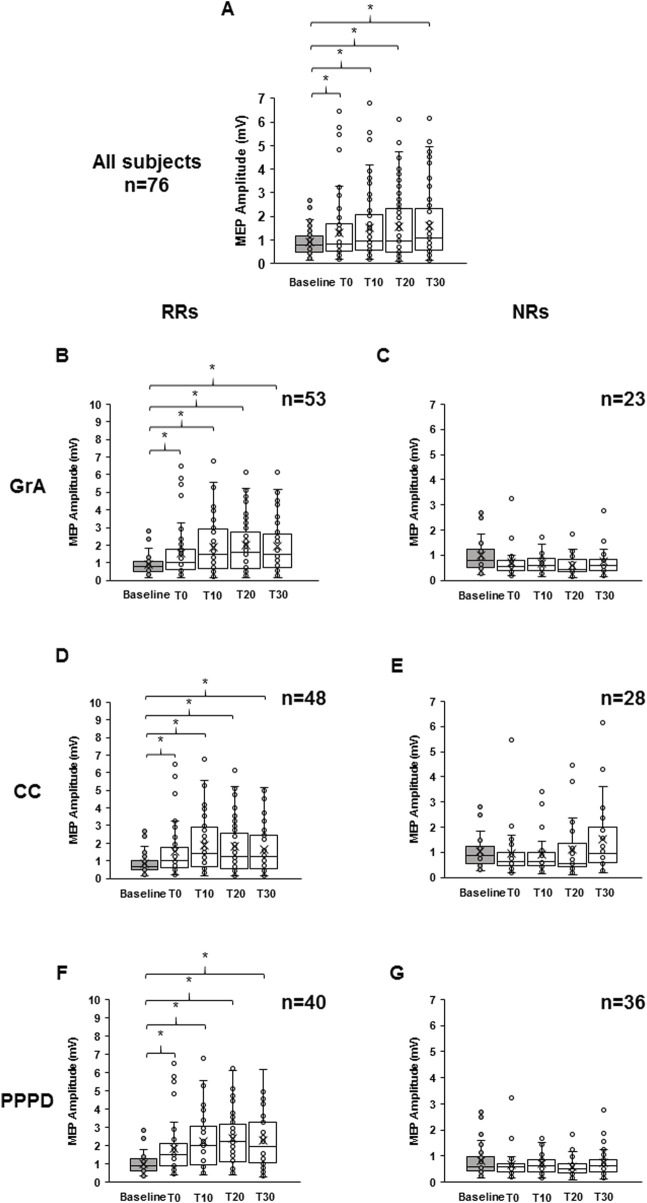
Effect of paired associative stimulation (PAS) on motor-evoked potentials (MEPs). The boxplots show MEP raw amplitudes at the different time points assessed: baseline (gray color), immediately after (T0), and after 10 min (T10), 20 min (T20), and 30 min (T30) from PAS delivery, for the all subjects **(A)**, for the responders **(B)**, and for the non-responders **(C)** to the PAS protocol. Responsiveness was determined using the grand average **(B, C)**, curve concavity **(D, E)**, and post-PAS difference **(F, G)**. The continuous line in the boxplots represents the median value, while the ‘ × ’ symbol represents the mean value of the group. Dots represent individual data. *p < 0.05.

When considering data from all participants, statistical analysis demonstrated a significant PAS effect at all time points tested. In particular, RM-one-way ANOVA showed a significant effect of time (F_4,72_ = 10.617, p < 0.001, η^2^p = 0.130), and Bonferroni’s *post hoc* analysis highlighted an increase in MEP amplitude compared to that at baseline at T0 (p = 0.040), T10 (p < 0.001), T20 (p < 0.001), and T30 (p < 0.001) (Figure 2A). When considering only the RR group for GrA categorization, RM-ANOVA detected a significant effect of time (F_4,49_ = 16.468, p < 0.001; η^2^p = 0.248), and *post hoc* analysis found that baseline MEP was significantly lower than MEPs at T0 (p = 0.003), T10 (p < 0.001), T20 (p < 0.001), and T30 (p < 0.001) (Figure 2B). On the other hand, when focusing the analysis on the NR group, the effect of time was again detected (F_4,19_ = 3.744, p = 0.031; η^2^p = 0.212), but *post hoc* analysis found that baseline MEP was significantly higher than that at T20 (p = 0.003), but not different when compared to that at T0 (p = 0.100), T10 (p = 0.072), and T30 (p = 0.261) (Figure 2C). When evaluating the RR group for CC categorization, the RM-ANOVA found significant effect of time (F_4,44_ = 14.107, p < 0.001; η^2^p = 0.243), and *post hoc* analysis found that the baseline MEP was significantly lower than MEPs at T0 (p = 0.002), T10 (p < 0.001), T20 (p < 0.001), and T30 (p = 0.001) (Figure 2D). When assessing NRs, a significant PAS effect was detected (F_4,24_ = 4.690, p = 0.002; η^2^p = 0.148), but *post hoc* analysis found no significant differences between baseline MEP and post-PAS time-points (all p > 0.05) (Figure 2E). Finally, when using PPPD as the discrimination PAS-index, a significant PAS effect for RRs was found (F 4.36 = 16.707, p < 0.001; η^2^p = 0.300). *Post hoc* analysis found that baseline MEP was significantly lower than MEPs at T0 (p = 0.003), T10 (p < 0.001), T20 (p < 0.001), and T30 (p < 0.001) (Figure 2F); however, when considering NRs for PPPD PAS-index, no significant PAS effect was detected (F_4,32_ = 1.741, p = 0.145; η^2^p = 0.052) (Figure 2G).

### 3.2 Evaluation of the characteristics of the PAS plasticity indexes

The curve estimate regression assessing the CC goodness of fit showed an *R* ^2^ of 0.212, with a mean RMSE of 0.674 mV. Linear regression analysis determined a significant goodness of fit between the modeled curve derived from both GrA and CC and the individual data obtained by the single time points (GrA: F_5,76_ = 61.855, p < 0.001; CC: F_5,76_ = 50,367.553, p < 0.001). When considering GrA, each independent variable significantly influenced it (baseline: standard β = −0.655, t71 = −10.380, p < 0.001; T0: standard β = −0.380, t71 = 4.194, p < 0.001; T10: standard β = −0.229, t71 = 2.357, p = 0.021; T20: standard β = −0.188, t71 = 1.587, p = 0.117; T30: standard β = 0.410, t71 = 4.677, p < 0.001), with the residual mean < 0.001 and a standard deviation of the residual of 0.454. When assessing CC, each independent variable significantly influenced the dependent one (baseline: standard β = 0.390, t71 = 159.457, p < 0.001; T0: standard β = −0.436, t71 = 127.950, p < 0.001; T10: standard β = −0.924, t71 = 246.332, p < 0.001; T20: standard β = −0.487, t71 = 105.845, p < 0.001; T30: standard β = 0.937, t71 = 273.573, p < 0.001), with the residual mean <0.001 and a standard deviation of the residual of 0.003.

The responsiveness rates and mean values of RMT and baseline MEPs for RRs and NRs groups based on each PAS plasticity index and the characteristics of the participants are shown in Table 1. Using GrA as the PAS plasticity index, 53 subjects (69.7%) were classified as RRs, and 23 subjects (30.3%) were classified as NRs. With CC, 48 subjects (63.2%) were identified as RRs, and 28 (36.8%) were identified as NRs, while PPPD classified 40 subjects (52.6%) as RRs and 36 (47.4%) as NRs. Seventeen subjects (22.4%) were consistently classified as RRs across all three indices, and 32 (42.1%) were consistently identified as NRs. Discrepancies in the responsiveness classification were observed between GrA and CC in five subjects (6.6%), between GrA and PPPD in 13 subjects (17.1%), and between CC and PPPD in eight subjects (10.5%). Linear regression modeling was utilized to evaluate the cortical excitability’s influence on MoCA and ACE-R scores, and it showed non-significant results (MoCA: F_2,74_ = 0.549, p = 0.580; ACE-R: F_2,74_ = 0.827, p = 0.441). This finding ruled out that cortical excitability could have influenced the results. One-way ANOVA failed to detect any significant difference in RMT and baseline MEP between RRs and NRs. In particular, one-way ANOVA showed non-significant effect of index when considering CC (RMT: F_1,75_ = 0.489, p = 0.487, η^2^p = 0.007; baseline MEP: F_1,75_ = 1.229, p = 0.271, η^2^p = 0.016), GrA (RMT: F_1,75_ = 0.122, p = 0.727, η^2^p = 0.002; baseline MEP: F_1,75_ = 0.345, p = 0.558, η^2^p = 0.005), and PPPD (RMT: F_1,75_ = 0.510, p = 0.477, η^2^p = 0.007; baseline MEP: F_1,75_ = 2.079, p = 0.153, η^2^p = 0.027). Chi-square analysis found that the RR rate was significantly higher than that of NRs when using GrA (Χ^2^_1,76_ = 11.842, p = 0.001, φ = 0.395) and CC (Χ^2^_1,76_ = 5.263, p = 0.022, φ = 0.266) as the PAS plasticity indexes, but it was not the same for PPPD (Χ^2^_1,76_ = 0.212, p = 0.646, φ = 0.053).

**TABLE 1:** Responsivity distribution and mean values of demographic features, baseline MEP, and RMT between RRs and NRs.

Plasticity indexes		CC	GrA	PPPD
Responsiveness (n)	RRs	48	53	40
	NRs	28	23	36
Age	RRs	67.4 ± 6.9	67.2 ± 7.4	67.1 ± 8.0
	NRs	66.5 ± 7.8	66.8 ± 6.7	67.0 ± 6.3
	p (ANOVA)	0.620	0.839	0.939
Sex	RRs	29 F–19 M	30 F–23 M	23 F–17 M
	NRs	12 F–16 M	11 F–12 M	18 F–18 M
	p (X2)	0.138	0.481	0.512
RMT (%MSO)	RRs	44.6 ± 7.7	45.1 ± 8.3	44.4 ± 8.1
	NRs	46.0 ± 9.7	44.6 ± 8.5	45.8 ± 8.9
	p (ANOVA)	0.487	0.727	0.477
Baseline MEP (mV)	RRs	0.9 ± 0.6	0.9 ± 0.5	1.0 ± 0.6
	NRs	1.0 ± 0.6	1.0 ± 0.7	0.8 ± 0.6
	*p(ANOVA)*	*0.271*	*0.558*	*0.153*

CC, curve concavity; GrA, grand average; PPPD, pre- vs. post-PAS difference; RRs, responders; NRs; non-responders; M, male; F female; RMT, resting motor threshold; %MSO, percentage of the maximum stimulator output; MEP, motor-evoked potential; *p(ANOVA)*, *p* value of the one-way ANOVA; *p(X* ^*2*^ *)*, *p* value of the chi-square test; *p < 0.05. Error measurement is represented by ± standard deviation.

ICC analysis found good consistency of CC, compared to GrA, in the description of the PAS-plasticity effect (ICC = 0.672, p < 0.001).

ORs to determine the likelihood to discriminate responsiveness, compared to GrA, found no differences between GrA and CC (OR = 0.744, p = 0.391); meanwhile, PPPD had significantly less likelihood to discriminate responsiveness than GrA (OR = 0.482, p = 0.032).

A contingency model using GrA as the reference index showed that CC had a sensibility ratio of 0.81 and a specificity of 0.63 in responsiveness discrimination, while PPPD demonstrated a sensibility ratio of 0.74 and a specificity of 1.0 (Table 2).

**TABLE 2:** Contingency table for subjects’ responsiveness to the PAS protocol when using CC or PPPD as the PAS plasticity index.

	Responsiveness to PAS	GrA	
		RRs	NRs
CC	RRs	42	6
	NRs	10	17
	Sensibility	42/(42 + 10) = 0.81	
	Specificity	17/(17 + 6) = 0.74	
PPPD	RRs	40	0
	NRs	13	23
	Sensibility	40/(40 + 13) = 0.75	
	Specificity	23/(23 + 0) = 1.0	

For both CC and PPPD panels: the top-left value identifies real RRs, the top-right value identifies false RRs, the bottom-left value identifies false NRs, and the bottom-right value identifies real NRs. Sensibility is calculated as [true RRs/(true RRs + false NRs)], while specificity is calculated as [true NRs/(true NRs + false RRs)].

GrA, grand average; RRs, responders; NRs, non-responders; CC, curve concavity; PPPD, pre- vs. post-PAS difference in MEP amplitude over at least two time-points.

When assessing the robustness of sensibility and specificity scores on a total of 54 selected subjects (70% of the whole sample), CC confirmed a good robustness for sensibility, while PPPD demonstrated good robustness for specificity. In fact, when using CC as the classification index, with GrA as the reference index, 35 subjects were considered as RRs and 19 as NRs. With this categorization, the sensibility accounted was 0.80, while the specificity was 0.20. When using PPPD as the index in comparison with GrA, the analysis showed that 31 subjects were considered as RRs and 23 as NRs, with a sensibility of 0.82 and a specificity of 1.00.

### 3.3 Assessment of the influence of gender and age on the neurophysiological tests and the PAS-plasticity indexes

Statistical analysis failed to detect any significant effect of gender on cognitive test scores (MMSE: F_1,75_ = 0.723, p = 0.398, η^2^p = 0.010; ACE-R: F_1,75_ = 2.701, p = 0.105, η^2^p = 0.035; MoCA: F_1,75_ = 0.290, p = 0.765, η^2^p = 0.001) and on PAS plasticity indexes (GrA: F_1,75_ < 0.00, p = 0.994, η^2^p < 0.001; CC: F_1,75_ = 1.145, p = 0.288, η^2^p = 0.015; PPPD: Χ^2^_1,76_ = 0.211, p = 0.646, φ = 0.075). Spearman’s analysis found no significant correlation between age and PAS plasticity indexes (GrA: r = 0.044, p = 0.079 and CC: r = −0.069, p = 0.551). When assessing the neuropsychological scores, MoCA and MMSE were not significantly correlated with age (MMSE: r = 0.082, p = 0.479; MoCA: r = 0.033, p = 0.776), while ACE-R showed a significant positive correlation with age (r = 0.370, p = 0.001). One-way ANOVA evaluation of age differences between RRs and NRs, using PPPD as the discriminating factor, found no significant difference (F_1,75_ = 0.006, p = 0.939).

### 3.4 Evaluation of the influence of responsiveness to the PAS protocol on the cognitive status

**FIGURE 3:**
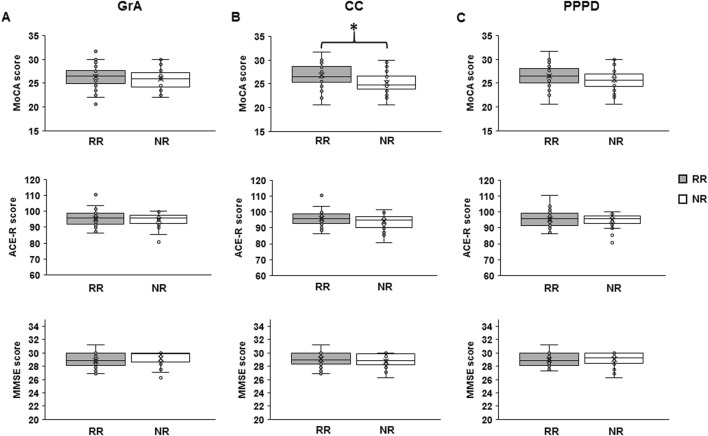
Differences in cognitive skills between responders (RRs) and non-responders (NRs) to the PAS protocol. The boxplots show the mean value of the Montreal Cognitive Assessment (MoCA), Addenbrooke’s Cognitive Examination revised (ACE-R), and Mini-Mental State Examination (MMSE) scores in RRs and NRs when using grand average (GrA) **(A)**, curve concavity (CC) **(B)**, and pre- vs. post-PAS difference (PPPD) **(C)** as the discrimination indexes for the responsiveness to PAS. The continuous line in the boxplots represents the median value, while the ‘ × ’ symbol represents the mean value of the group. Dots represent individual data. *p < 0.05.

Table 3 shows the mean values of the MMSE, ACE-R, and MoCA scores for the RR and NR groups based on each PAS-plasticity index. One-way ANOVA showed that MMSE and ACE-R scores were not significantly different between RRs and NRs when considering GrA (MMSE: F_1,75_ = 0.870, p = 0.354, η^2^p = 0.012; ACE-R: F_1,75_ = 0.764, p = 0.385, η^2^p = 0.010), CC (MMSE: F_1,75_ = 1.232, p = 0.271, η^2^p = 0.016; ACE-R: F_1,75_ = 3.315, p = 0.073; η^2^p = 0.043), and PPPD (MMSE: F_1,75_ = 0.175, p = 0.677, η^2^p = 0.002; ACE-R: F_1,75_ = 0.469, p = 0.496, η^2^p = 0.006). A non-significant difference in the MoCA score was found between RRs and NRs for GrA (F_1,75_ = 0.234, p = 0.630, η^2^p = 0.003) and PPPD (F_1,75_ = 1.699, p = 0.196, η^2^p = 0.022); conversely, RRs showed a higher MoCA score than NRs when CC was used as the PAS-plasticity index (F_1,75_ = 7.963, p = 0.006, η^2^p = 0.097) (Figure 3).

**TABLE 3:** Mean values and statistical differences of the Mini-Mental State Examination, Addenbrooke’s Cognitive Examination revised, and Montreal Cognitive Assessment between RRs and NRs.

Cognitive score		GrA			CC			PPPD		
Cognitive test	Categories	RRs	NRs	*p*	RRs	NRs	*p*	RRs	NRs	*p*
MMSE		28.9 ± 1.0	29.1 ± 1.1	*0.354*	29.1 ± 1.1	28.8 ± 1.1	*0.271*	28.9 ± 1.0	29.0 ± 1.1	*0.677*
ACE-R	Total score	95.5 ± 4.7	94.5 ± 4.7	*0.385*	95.6 ± 3.8	93.9 ± 5.1	*0.106*	95.5 ± 5.1	94.8 ± 4.2	*0.496*
	Orientation	18.0 ± 0.3	18.0 ± 0.2	*0.783*	18.0 ± 0.2	18.0 ± 0.2	*0.216*	18.0 ± 0.3	18.1 ± 0.2	*0.435*
	Memory	23.7 ± 2.9	22.4 ± 4.1	*0.122*	24.0 ± 2.6	22.0 ± 4.0	0.009*	23.5 ± 2.9	23.1 ± 3.8	*0.604*
	Verbal fluency	11.7 ± 2.0	11.9 ± 1.7	*0.635*	11.6 ± 2.1	12.0 ± 1.6	*0.353*	11.7 ± 2.0	11.9 ± 1.8	*0.644*
	Language	26.2 ± 1.0	26.3 ± 0.7	*0.745*	26.2 ± 0.9	26.4 ± 0.9	*0.319*	26.2 ± 1.1	26.3 ± 0.7	*0.609*
	Visuo-spatial	15.4 ± 1.3	15.4 ± 1.1	*0.993*	15.5 ± 1.3	15.3 ± 1.2	*0.595*	15.6 ± 1.2	15.3 ± 1.3	*0.300*
MoCA	Total score	26.3 ± 2.5	26.0 ± 2.5	*0.630*	26.8 ± 2.4	25.2 ± 2.3	0.006*	26.6 ± 2.4	25.8 ± 2.5	*0.196*
	Visuo-spatial	3.6 ± 0.6	3.8 ± 0.4	*0.201*	3.7 ± 0.7	3.6 ± 0.5	*0.615*	3.7 ± 0.7	3.7 ± 0.5	*0.814*
	Executive functions	3.5 ± 0.6	3.4 ± 0.75	*0.608*	3.5 ± 0.6	3.4 ± 0.8	*0.636*	3.5 ± 0.6	3.4 ± 0.7	*0.531*
	Language	5.6 ± 0.6	5.6 ± 0.7	*0.889*	5.7 ± 0.6	5.4 ± 0.8	*0.120*	5.6 ± 0.7	5.5 ± 0.7	*0.649*
	Orientation	6.0 ± 0.27	6.0 ± 0.1	*0.477*	6.0 ± 0.3	6.0 ± 0.1	*0.392*	6.0 ± 0.3	6.0 ± 0.1	*0.328*
	Attention	5.6 ± 0.6	5.6 ± 0.6	*0.764*	5.7 ± 0.6	5.5 ± 0.6	*0.183*	5.7 ± 0.6	5.6 ± 0.6	*0.492*
	Memory	2.6 ± 1.6	2.2 ± 1.7	*0.312*	2.79 ± 1.5	2.0 ± 1.6	0.036*	2.7 ± 1.5	2.3 ± 1.7	*0.317*

GrA, grand average; CC, curve concavity; PPPD, pre vs. post-PAS difference; RRs, responders; NRs; non-responders; MMSE, Mini-Mental State Examination; MoCA, Montreal Cognitive Assessment; ACE-R, Addenbrooke’s Cognitive Examination revised; *p*, p-value of the one-way ANOVA; *p < 0.05. Error measurement is represented by ± standard deviation.

Analysis of the subcategories of MoCA and ACE-R showed a significant difference between RRs and NRs for the three PAS indexes (GrA, CC, and PPPD) in the MoCA and ACE-R subcategory of memory, but it was only when CC was used as the discriminative index (MoCA: F_1,75_ = 4.559, p = 0.036, η^2^p = 0.058; ACE-R: F_1,75_ = 7.212, p = 0.009, η^2^p = 0.089) (Table 3).

Bayesian analysis confirmed the ANOVA results. In fact, when using GrA and PPPD as the discriminative PAS plasticity indexes, BF always showed moderate evidence to accept the null hypothesis (GrA: MMSE BF = 0.139, MoCA BF = 0.101, ACE-R BF = 0.114; PPPD: MMSE BF = 0.099, MoCA BF = 0.207, ACE-R BF = 0.114); when using CC, it showed moderate evidence for acceptance of the null hypothesis for MMSE (BF = 0.165), anecdotal evidence for acceptance of the null hypothesis for ACE-R (BF = 0.499), but moderate evidence for acceptance of the alternative hypothesis for MoCA (BF = 3.794). The same results have been found for the cognitive subcategories: moderate evidence for acceptance of the null hypothesis when using both GrA and PPPD for all subcategories; meanwhile, when using CC, the memory subcategory of ACE-R showed anecdotal evidence for acceptance of the alternative hypothesis (BF = 2.709), and the memory subcategory of MoCA showed anecdotal evidence for acceptance of the null hypothesis (BF = 0.804); the other subcategories of ACE-R and MoCA showed moderate evidence for acceptance of the null hypothesis.

Table 4 reports demographic, neurophysiological and cognitive values divided by sample and responsiveness to the PAS protocol.

**TABLE 4:** Demographic, neurophysiological, and cognitive values of the sample by decade and responsiveness to the PAS protocol.

	Decade	Responsiveness	Numerosity	Sex	RMT	Baseline MEP	MMSE score	MoCA score	ACE-R score
GrA	50–59	RRs	9	6 F–3 M	43.3 ± 7.1	0.9 ± 0.6	28.6 ± 0.8	25.7 ± 1.9	91.9 ± 4.2
		NRs	4	4 M	39.0 ± 2.6	1.2 ± 0.4	28.4 ± 1.6	26.1 ± 2.9	91.2 ± 5.9
	60–69	RRs	24	13 F–11 M	45.5 ± 8.4	0.8 ± 0.4	29.0 ± 1.0	26.8 ± 2.2	95.3 ± 3.5
		NRs	11	3 F–8 M	45.4 ± 7.1	1.0 ± 0.7	29.4 ± 1.0	25.5 ± 2.4	94.3 ± 5.2
	70–79	RRs	17	10 F–7 M	46.0 ± 9.5	0.9 ± 0.6	28.8 ± 1.3	26.2 ± 3.2	96.2 ± 4.5
		NRs	7	4 F–3 M	43.4 ± 8.8	0.9 ± 0.7	29.5 ± 0.6	27.1 ± 2.2	96.2 ± 3.3
	80–89	RRs	3	1 F–2 M	46.0 ± 10.0	1.1 ± 0.4	29.2 ± 1.1	25.5 ± 1.0	103.2 ± 6.7
		NRs	1	M	66.0	0.45	27.1	22.5	94.6
CC	50–59	RRs	9	7 F–2 M	44.0 ± 6.4	0.9 ± 0.6	28.8 ± 0.9	26.6 ± 1.9	93.3 ± 4.5
		NRs	4	3 F–1 M	37.5 ± 3.4	1.2 ± 0.4	28.0 ± 1.2	24.3 ± 1.7	88.8 ± 3.0
	60–69	RRs	21	12 F–9 M	45.9 ± 7.8	0.8 ± 0.6	29.3 ± 0.9	26.9 ± 1.1	95.7 ± 3.2
		NRs	14	4 F–10 M	44.9 ± 8.4	0.8 ± 0.4	28.9 ± 1.1	25.6 ± 2.3	93.9 ± 5.0
	70–79	RRs	16	10 F–6 M	42.2 ± 8.0	0.7 ± 0.4	29.0 ± 1.3	27.2 ± 3.0	96.6 ± 3.8
		NRs	8	4 F–4 M	50.9 ± 9.0	1.3 ± 1.0	29.1 ± 0.8	25.2 ± 2.7	95.4 ± 4.7
	80–89	RRs	2	2 M	51.0 ± 7.0	1.0 ± 0.5	29.2 ± 1.6	25.5 ± 1.4	104.1 ± 9.1
		NRs	2	1 F–1 M	51.0 ± 21.2	0.9 ± 0.6	28.1 ± 1.4	24.0 ± 2.1	98.0 ± 4.9
PPPD	50–59	RRs	7	6 F–1 M	42.6 ± 8.0	1.0 ± 0.7	28.3 ± 0.6	25.5 ± 2.0	90.8 ± 3.7
		NRs	6	4 F–2 M	41.3 ± 4.3	1.0 ± 0.5	28.8 ± 1.4	26.3 ± 2.3	93.2 ± 5.4
	60–69	RRs	20	11 F–9 M	45.4 ± 8.5	0.8 ± 0.4	29.0 ± 1.0	27.0 ± 2.1	95.3 ± 3.8
		NRs	15	5 F–10 M	45.5 ± 7.5	0.9 ± 0.7	29.3 ± 0.9	25.6 ± 2.4	94.6 ± 4.5
	70–79	RRs	10	5 F–5 M	43.1 ± 7.6	1.2 ± 0.7	29.2 ± 1.2	27.0 ± 3.4	96.9 ± 4.9
		NRs	14	9 F–5 M	46.6 ± 10.1	0.7 ± 0.6	29.0 ± 1.1	26.1 ± 2.7	95.7 ± 3.5
	80–89	RRs	3	1 F–2 M	46.0 ± 10.0	1.1 ± 0.4	29.2 ± 1.1	25.5 ± 1.0	103.2 ± 6.7
		NRs	1	M	66.0	0.45	27.1	22.5	94.6

RMT, resting motor threshold; MEP, motor-evoked potential; MMSE, Mini-Mental State Examination; MoCA, Montreal Cognitive Assessment; ACE-R, Addenbrooke’s Cognitive Examination revised; GrA, grand average; CC, curve concavity; PPPD, pre vs. post-PAS difference; RRs, responders; NRs; non-responders. Error measurement is represented by ± standard deviation.

### 3.5 Assessment of the correlation between PAS plasticity indexes and cognitive status

**FIGURE 4:**
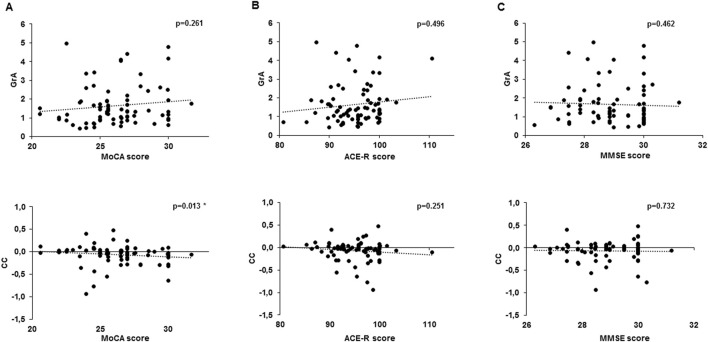
Correlation analysis between cognitive test scores and PAS plasticity indexes. The chart shows a correlation between the Montreal Cognitive Assessment (MoCA) **(A)**, Addenbrooke’s Cognitive Examination revised (ACE-R) **(B)**, and Mini-Mental State Examination (MMSE) **(C)** with the grand average (GrA) (upper line) and curve concavity (CC) (bottom line). Dotted lines express the trending line of the correlation. *p < 0.05.

Normality tests indicated that the variables included in the correlation analysis were not normally distributed: GrA (K–S = 0.167, p < 0.001; S–W = 0.840, p < 0.001), CC (K–S = 0.191, p < 0.001; S–W = 0.861, p < 0.001), MMSE (K–S = 0.193, p < 0.001; S–W = 0.918, p < 0.001), MoCA (K–S = 0.099, p = 0.063; S–W = 0.973, p = 0.104), and ACE-R (K–S = 0.103, p = 0.045; S–W = 0.968, p = 0.055). Consequently, Spearman’s rank test was applied. Spearman’s correlation analysis detected a significant negative correlation between MoCA and CC (r = −0.285, p = 0.013) but not between MoCA and GrA (r = 0.131, p = 0.261). ACE-R and MMSE were not correlated with either CC (MMSE: r = −0.040, p = 0.732; ACE-R: r = −0.134, p = 0.251) or GrA (MMSE: r = −0.086, p = 0.462; ACE-R: r = 0.080, p = 0.496) (Figure 4). No significant correlations were detected between the two PAS plasticity indexes (GrA and CC) and the subcategories of MoCA and ACE-R.

## 4 Discussion

This work analyzed, for the first time, different plasticity indexes that can reflect the PAS-induced LTP phenomena and the cognitive status of aged, healthy subjects. Overall, the results of the present study showed that CC is the PAS plasticity index that is able to better discriminate between RRs and NRs, as demonstrated by its consistency, high ORs, and sensibility in the discrimination of RRs and NRs to the PAS protocol.

In addition, CC was the only PAS plasticity index that was able to describe the cognitive status in a group of aged, healthy subjects, as demonstrated by its significant correlation with the MoCA score, which was, in turn, significantly different between RRs and NRs.

Notably, LTP is a key mechanism of synaptic plasticity, where specifically stimulated synaptic connections are strengthened following high-frequency stimulation and near-synchronous activation of both pre- and post-synaptic neurons (Malenka and Bear, 2004; Kandel et al., 2014). PAS is widely used in humans to study LTP at the cortical level, and it should determine a rapidly evolving (<30 min) and long-lasting (>60 min) increase in cortical excitability, resulting in an increase in MEP amplitude (Stefan et al., 2000; 2002). Due to its features, LTP–PAS effects should be observed as a long-lasting and non-isolated phenomenon (Malenka and Bear, 2004; Kandel et al., 2014), as defined by a curve-shaped trend, determined by a transient initial increment in the cortical excitability, until it returns to baseline levels. According to this point of view, that GrA is the most-used PAS-plasticity index in literature could be the result of an increase of M1 excitability rather than an LTP phenomenon. In fact, GrA is determined by the mean value of the MEP obtained at post-PAS time points (Wolters et al., 2005; Müller-Dahlhaus et al., 2008; Lahr et al., 2016; Minkova et al., 2019), which strongly influence it. For instance, for a substantial increase in MEP amplitude in only one time point, it may strongly influence the final GrA value. In addition, MEP changes at a single time point could reflect a simple change in cortical excitability rather than a plastic effect in M1 (Stefan et al., 2000). The calculation of PPPD and CC considered this point of view. In fact, PPPD discriminates RRs only when at least two out of the four post-PAS MEP amplitudes were significantly higher than baseline MEP values, thus individuating RRs only if the change in MEP amplitude was consistently present in two time points, which is in agreement with the existence of a long-lasting phenomenon. This was also supported by the high specificity shown in the contingency table model (Table 2), where GrA was used as a reference index since it is the most widely used index of PAS plasticity in literature (Wolters et al., 2005; Müller-Dahlhaus et al., 2008; Lahr et al., 2016; Minkova et al., 2019). Along the same line, CC was able to well-describe the LTP–PAS effects. In particular, CC was calculated through the polynomial function of the curve obtained from the MEP amplitudes assessed in all the five time points considered. A negative value (negative concavity) identified RRs, while a positive value (positive concavity) determined NRs. Due to its mathematical characteristics, CC describes the whole phenomenon. Therefore, to produce a negative concavity, the phenomenon should be long-lasting and not dependent on an isolated change in MEP amplitude. This observation was also supported by the high sensibility of CC (Table 2) that, although showing less specificity than PPPD, it had higher sensibility.

Notably, when dealing with the contingency model, sensibility identifies the ability of an index to more accurately discriminate subjects that do not respond to the PAS protocol; meanwhile, specificity describes the index characteristic to better discriminate the RRs to the PAS protocol (Zweig and Campbell, 1993; Kallner, 2018). The analysis showed that the PPPD index has a high specificity since it identifies fewer RRs than CC and GrA (Table 1), determining the increased accuracy in the identification of the RRs in reference to GrA but less accuracy for the identification of NRs, as determined by the reduced OR compared to that of GrA. In contrast, CC demonstrated a higher sensibility than PPPD, with no ORs difference compared to that of GrA, suggesting its good capability in the correct discrimination of the NRs. Therefore, it is likely that the most accurate identification of RRs may be made by PPPD, as determined by the few false RRs in the contingency table (Table 2) and the analysis of robustness, while CC is able to better discriminate the NRs as GrA, as shown by few false NRs (Table 2) and the analysis of robustness. Although both PPPD and CC differently discriminated responsiveness to the PAS protocol and described the LTP phenomenon well, these indexes rely on MEP recordings from at least three time points, which could be time-consuming. Compared to GrA and PPPD, CC appears to be a better index to describe cognitive status, as demonstrated by the correlation with the MoCA score and the difference in MoCA between RRs and NRs.

Overall, the CC and PPPD PAS plasticity indexes can serve as reliable measures of PAS-induced plasticity across different contexts.

Based on the calculation of the ICC and OR, it is likely that CC is consistent in the assessment of PAS effects at least as much as GrA, and it is useful when the investigation is focused on the whole long-lasting plastic phenomenon. In fact, this can help not only to determine whether the PAS effects occurred or not but also its occurrence in a long-lasting post-PAS period. Moreover, in light of the relation found between CC and cognitive scores, this index appears to also be useful in studies that would correlate neurophysiological and cognitive aspects. The curve estimate regression model detected a weak goodness of fit between raw MEPs and the curve underpinning CC. The low R2 can likely be attributed to the intrinsic inter- and intra-subject variability of the MEP amplitude, which reflects fluctuations in cortical excitability during the experiments and, thus, requires several measure repetitions (Rossini et al., 2015).

On the other hand, the high specificity of PPPD makes it perfect for finding RRs with high reliability, even if it results in fewer RRs than with other indexes. This may be useful in studies with large samples, allowing analyses that are only focused on RRs, and for evaluating few post-PAS time points (at least two).

As mentioned before, compared to GrA and PPPD, CC seems to be a better index to describe the cognitive status, as demonstrated by the correlation with the MoCA score and the difference in MoCA between RRs and NRs. This observation is in contrast with those of previous studies that failed to detect any significant difference in cognitive status between RRs and NRs (Schättin et al., 2018; Minkova et al., 2019). These studies used the GrA, which did not correlate with cognitive status in our study as well. The significant correlation observed between CC and MoCA can be explained by CC characteristics, describing a curve-shaped trend of the long-lasting PAS plasticity effect, differently from the GrA. This finding suggests that CC may represent a useful index to investigate PAS-induced plastic events in humans in relation to cognitive scores, at least in healthy, aged subjects.

It is well known that synaptic plasticity is strongly connected to cognitive status. Previous studies found that cognitive decline is associated with altered connectivity between brain areas and reduced synaptic plasticity (Bassi et al., 2019). Moreover, it has been previously suggested that cognitive reserve may be intimately related to cortical excitability and cortical plasticity (Freitas et al., 2013; Palermo et al., 2025). Cognitive reserve allows cognitive functions to be maintained—or minimally impaired—in the elderly population and can enable individuals to sustain more neuropathological insults before they manifest cognitive decline (Freitas et al., 2013). It has been hypothesized that the gradual change in the relationship between altered cortical excitability and cognitive performance reflects the point at which hyperexcitability becomes compensatory and detrimental to individuals experiencing cognitive impairments, related to an increasing impediment in the allocation of cognitive resources (Palermo et al., 2025). The cognitive reserve serves to prolong functioning and delay the reaching of this critical point, additionally influencing the magnitude of plastic changes. For example, in patients with mild Alzheimer’s disease (AD) with the same degree of cognitive decline, highly educated patients (higher cognitive reserve) have less advanced pathological and functional brain changes (Kemppainen et al., 2008), which suggests that the clinical manifestation of advanced AD pathology is delayed in individuals with higher educational attainment. Adaptive (or compensatory) network plasticity might, thus, represent the neurobiological substrate of cognitive reserve, and our results are in line with those of these studies, suggesting CC as a potential plasticity index to possibly assess cognitive reserve both in pathological and healthy aging populations.

In the present study, aged subjects were investigated, which rules out a possible ceiling effect in the results of neuropsychological tests when performed by young people. However, the lack of difference in ACE-R and MMSE scores between RRs and NRs suggests that the difference found when using MoCA to discriminate responsiveness to PAS could be because of the different types of the neuropsychological tests used. Previous findings observed that among the tests that are useful to discriminate the cognitive status between healthy and pathological subjects, MoCA is the most reliable and sensible one (Nasreddine et al., 2005). For this reason, it could be suggested that total ACE-R and MMSE failed to be associated with cortical plasticity and discriminate responsiveness to PAS because of their lower sensitivity than MoCA. Finally, RRs had higher memory skills than NRs, as demonstrated by MoCA and ACE-R subcategory analysis, only when using CC as the discriminative PAS-plasticity index. This result is in line with the literature describing how plasticity phenomena are the mechanisms underlining memory function. In this light, CC may strongly represent a key tool to investigate the relation between cognitive functions and plasticity in humans.

However, it has to be noted that responsiveness to a plasticity protocol could be influenced by several factors such as alertness (Kamke et al., 2012), attentional state (Stefan et al., 2004), stimulation intensity (Müller-Dahlhaus et al., 2008), genetic traits (Cheeran et al., 2008; Missitzi et al., 2011; Peña-Gomez et al., 2012; Fried et al., 2017), cortical thickness (List et al., 2013), and microstructural properties of white matter (Klöppel et al., 2008). Previous studies focusing on genetic factors found that polymorphism of the brain-derived neurotrophic factor gene (BDNF) (Cheeran et al., 2008; Fried et al., 2017) discriminated between RRs and NRs to repetitive TMS. BDNF has a variety of roles in cognition (Velioglu et al., 2021), and it has been shown to modulate NMDAR-dependent LTP in animal models (Figurov et al., 1996). Although a previous study failed to find a relation between BDNF polymorphism and PAS responsiveness (Minkova et al., 2019), future studies may try to relate the frequency of BDNF polymorphism with PAS responsiveness using CC as the PAS plasticity index.

The categorization and comparison of responsiveness to the PAS protocol across studies remain challenging, largely because of the heterogeneity of the protocols and their analysis in the existing literature. Future studies should focus on filling this gap by incorporating multiple post-PAS assessment time points, selecting PAS-induced plasticity indices that are best aligned with the specific study objectives, and increasing the sample size. These methodological features would help to minimize the influence of NRs on the overall findings, thereby allowing a more targeted investigation of RRs and potentially providing insights into PAS responsiveness in the context of cognitive decline.

### 4.1 Limitation of the study

We acknowledge that this study has some limitations. First, the study is not sham-controlled due to the absence of a true PAS-sham protocol available in the literature. However, future studies should reach a consensus on standardized protocols to more effectively assess PAS effects.

Additionally, a real gold standard for synaptic plasticity assessment is not available for comparison and to calculate the specificity and sensibility of the new PAS indexes. In fact, the comparison in terms of sensibility and selectivity between CC and PPPD as PAS plasticity indexes is referenced to GrA (the most used in literature), which does not represent a real observation of plastic changes in the brain. Without an external or clinical benchmark, the current results largely reflect the internal consistency between metrics.

In the present study, PAS effects have been investigated until 30 min after paired stimuli delivery. However, because of the high inter- and intra-subject variability, it is possible that some subjects may respond to the protocol after this time window. Hence, it appears to be worthwhile for future studies to investigate responsiveness to PAS after 30 min in subjects who do not respond in the first 30 min. Finally, future studies should investigate how some genetic and anatomical features of the subjects may influence responsiveness to the PAS protocol and the magnitude of the PAS-effect.

## 5 Conclusion

In conclusion, CC may represent a potential PAS-plasticity index that is useful for describing cortical plasticity and cognitive skills in humans, with a possible practical application in patients with cognitive impairment, namely, Alzheimer’s disease.

## Data Availability

The data that support the findings of this study are made available from the corresponding author, upon reasonable request.
